# Olanzapine Promotes the Occurrence of Metabolic Disorders in Conditional TCF7L2-Knockout Mice

**DOI:** 10.3389/fcell.2022.890472

**Published:** 2022-07-06

**Authors:** Ye Yang, Manjun Shen, Li Li, Yujun Long, Lu Wang, Bing Lang, Renrong Wu

**Affiliations:** ^1^ National Clinical Research Center for Mental Disorders, Department of Psychiatry, The Second Xiangya Hospital of Central South University, Changsha, China; ^2^ Shenzhen Nanshan Center for Chronic Disease Control, Department of Psychiatry, Shenzhen, China

**Keywords:** TCF7L2, CKO, olanzapine, metabolic disorders, GLP-1R

## Abstract

**Objectives:** Schizophrenia (SCZ) patients display higher incidence of metabolic syndrome (MetS) and comorbidity of type II diabetes. Both atypical antipsychotics and genetic variants are believed to predispose the patients with the risk, but their interplay remains largely unknown. TCF7L2 is one of the most common genes strongly associated with glucose homeostasis which also participates in the pathogenesis of schizophrenia. In this study, we aimed to explore the regulatory roles of TCF7L2 in atypical antipsychotics-induced MetS.

**Methods:** Mice with pancreatic β-cell–specific Tcf7l2 deletion (CKO) were generated. The CKO mice and control littermates were subjected to olanzapine (4 mg/kg/day) or saline gavage for 6 weeks. Metabolic indices, β cell mass, and the expressing levels of TCF7L2 and GLP-1R in the pancreatic tissue were closely monitored.

**Results:** Tcf7l2 CKO mice displayed a spectrum of core features of MetS, which included remarkably increased rate of weight gain, higher fasting insulin, higher values of blood lipids (cholesterol, triglyceride, and low-density lipoprotein), impaired glucose tolerance, and hypertrophy of adipocytes. Notably, these effects could be further exacerbated by olanzapine. In addition, Tcf7l2 CKO mice with the olanzapine group showed significantly decreased expressions of GLP-1R protein and a trend of reduced pancreatic β-cell mass. RT-qPCR revealed that the CKO mice presented a significantly less transcription of Sp5, an important element of the Wnt signaling pathway.

**Conclusion:** Our study illustrates that mice with pancreatic β-cell–targeted Tcf7l2 deletion were more vulnerable to suffer metabolic abnormalities after olanzapine administration. This impairment may be mediated by the reduced expression of GLP-1R.

## Introduction

Schizophrenia (SCZ) is a chronically disabling brain disorder which affects around 1% of the global population (Stilo and Murray, 2010). Despite the strenuous research in the past several decades, SCZ still remains largely elusive. Both genetic and environmental factors are implicated, and the hereditary is high (60%–80%) (Khavari and Cairns, 2020); however, the precise genetic landscape and environmental interaction mechanism remain incredibly understudied. As a result, SCZ patients suffer a considerably short life expectancy compared with the general population (Correll et al., 2017). Amounting epidemiological evidence has suggested a high occurrence of metabolic syndrome (MetS) which strongly contributes to the shorter life span presented by SCZ patients (Jayatilleke et al., 2017). MetS features glucose intolerance, insulin resistance, dyslipidemia, type 2 diabetes mellitus (T2DM), and obesity which predispose to an excess cardiovascular mortality. In a recent review, individuals with schizophrenia concomitant diabetes ranged from 1.26% to 50% across studies, with median 13% (Ward and Druss, 2015), highlighting a high comorbidity of diabetes and SCZ. Intriguingly, SCZ patients with untreated diabetes showed poorer overall cognitive performance (Takayanagi et al., 2012), which could be effectively ameliorated by anti-diabetic drugs such as metformin (Wu et al., 2008). Meanwhile, patients diagnosed with diabetes are more likely to exhibit cognition impairment and higher risk to develop major mental illnesses such as SCZ or bipolar disorder. As such, a bi-directional promotion model has been proposed, and existence of common pathophysiological mediators in both diabetes and schizophrenia has been hypothesized (Takayanagi et al., 2020).

It is considerably arguable to identify the common mediators to the comorbidity of MetS and SCZ. The widely used atypical antipsychotics (APPs) often associate with high prevalence of metabolic disorders (35.3%) in patients with schizophrenia, indicating a critical role of APPs in the comorbidity of MetS in SCZ patients (Jeon and Kim, 2017). Olanzapine is one of the most widely used APPs in clinics with established effects of weight gain and severe MetS, which has been well demonstrated by meta-analyses (Huhn et al., 2019), randomized clinical trials (RCTs) (McEvoy et al., 2007), and animal studies (Coccurello et al., 2006). Largely due to these unpleasant adverse effects, the withdrawal rate of olanzapine is 2–8 times higher than that of other antipsychotics (Lieberman et al., 2005).

Apart from the APPs, the polygenic nature of schizophrenia also predisposes patients with the higher risk of the onset of MetS. Glucose intolerance is one of the core features of MetS which, however, is frequently reported in drug-naïve psychotic patients including SCZ (Fernandez-Egea et al., 2009; Garcia-Rizo et al., 2016; Greenhalgh et al., 2017). This strongly indicates that the higher incidence of MetS in SCZ patients also originates from intrinsic vulnerability to metabolic disturbance. However, up to date, there is almost no literature report to describe the interaction between genetic variants and APP in SCZ patients or the underlying mechanism.

TCF7L2 is a Wnt signaling-associated transcription factor which is strongly expressed in several tissues, especially the pancreas. It can stimulate the pancreatic β-cells’ proliferation (Jin, 2016) *via* Wnt signaling (Yi et al., 2005) and stands as one of the best-established candidate genes for type 2 diabetes (Lyssenko et al., 2007). Decreased TCF7L2 protein closely correlates with downregulated GIP- and GLP-1 receptors, two key determinants for β-cells’ proliferation and survival, as well as glucose-stimulated insulin secretion (Shu et al., 2009a). Intriguingly, recent genetic data have demonstrated TCF7L2 polymorphisms as high-risk alleles for human SCZ across world population (Hansen et al., 2011; Alkelai et al., 2012; Liu et al., 2017). In mice, manipulation of the Tcf7l2 expression through gain/loss-of-function approaches leads to anxiety-like behavior and dosage-dependent contextual fear learning impairment (Savic et al., 2011). Deletion of Tcf7l2 across the pancreatic tissue (da Silva Xavier et al., 2012) or specifically in β-cells disrupts the healthy glucose tolerance and insulin secretion (Mitchell et al., 2015). Thus, TCF7L2 has been hypothesized as an important risk gene co-shared by type 2 diabetes, MetS, and psychiatric disorders, especially SCZ (Postolache et al., 2019).

While the aforementioned evidence suggests that TCF7L2 mutation may impair proper pancreatic function and cognition, there is scarce literature deciphering the regulatory roles of TCF7L2 in APP-induced MetS. Previously, we have shown that olanzapine challenge associates an increased expression of Tcf7l2 in non-pancreatic tissues and concomitant dyslipidemia (Li et al., 2018), highlighting an active interaction between olanzapine and Tcf7l2. In the present study, we further addressed this question using pancreatic β-cell–specific Tcf7l2-knockout mice. Our results support a central role of TCF7L2 in β cells for olanzapine-induced metabolic abnormalities and when disrupted, promote the progress of MetS.

## Materials and Methods

### Animals

**FIGURE 1:**
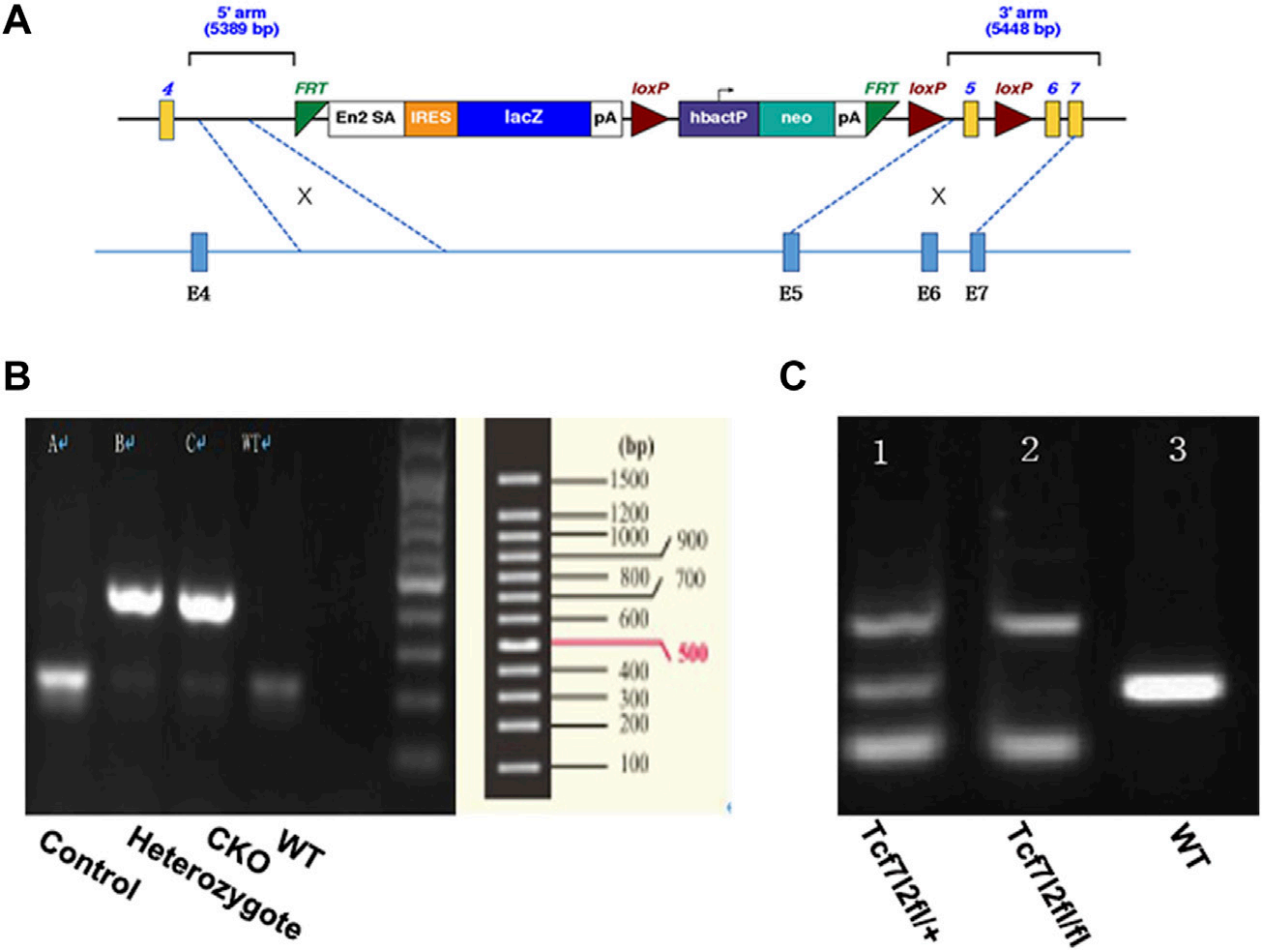
Generation of mice with pancreatic β-cell–targeted Tcf7l2 deletion. **(A)** Diagram of the construct used to generate Tcf7l2^fl/fl^ mice. It should be noted that exon 5 was flanked by two loxp sites and was supposed to be deleted at the presence of cre-recombinase. **(B)** PCR results showed mice with cre-recombinase (400 bp, lanes 2 and 3) or littermate control (endogenous band at 200 bp, lane 1). The sample at lane 4 was from a C57 mouse as a blank control. **(C)** PCR results showed genotypes of Tcf7l2^fl/+^ (lane 1, with the PCR products at 139 bp, 281 bp, and 500 bp) and Tcf7l2^fl/fl^ mice (lane 2, with the PCR products at 139 bp and 500 bp). Lane 3 displayed the PCR product (281 bp, endogenous band) from a C57 mouse as a blank control.

The Tcf7l2^tm1a(EUCOMM)Wtsi^-knockout-first mice were purchased from the European Mouse Mutant Archive (https://www.infrafrontier.eu/procedures/animal-welfare-and-ethics/emma-repository). The knock-out-first construct contained the sequence of FRT-En2SA-IRES-LacZ-PolyA-loxP-hbactP-neo-PolyA-FRT-loxP and was used to target sequences of intron 5. A further loxP was introduced prior to exon 6, which enables exon 5 flanked by two loxp insertions and the potential to create conditional Tcf7l2-knockout mice if required. The detailed vector diagram is shown in Figure 1A. The Tcf7l2^tm1a(EUCOMM)Wtsi^-knockout-first mice were crossed with Flp transgenic mice in order to remove the sequence between the two FRT sites and to create Tcf7l2^fl/fl^ mice. To specifically delete Tcf7l2 in β cells of the pancreas, Tcf7l2^fl/fl^ mice were crossed with Ins2-Cre mice to generate Tcf7l2^fl/fl^: In2-cre (referred as Tcf7l2 CKO) mice. Both the Ins2-Cre and Flp mice were obtained from the Shanghai Model Organisms Center. All the mice were maintained on a C57BL/6 background with free access to regular chow and water. An average of three to five animals was housed per cage under a 12 h light-dark cycle. All experiments were performed in accordance with the Guidelines and Regulation of Laboratory Animals Used for Biomedical Studies of Xiangya Second Hospital, China. Animal care practices and all experiments were reviewed and approved by the Animal Committee of Xiangya Second Hospital, Central South University, Changsha, China.

Tcf7l2 CKO mice and control littermates were obtained from mating of Tcf7l2^fl/fl^: In2-cre mice to achieve Tcf7l2^fl/fl^ and In2-cre (referred as CKO) and Tcf712^fl/fl^ as controls. Mice were born at the expected Mendelian ratios with no apparent abnormalities. Each mouse was genotyped using genomic DNA with pairs of primers listed in Table 1 Two bands (139 bp and 500 bp) can be detected for mice with successful exon 5 deletion, while a 281 bp band will be amplified for WT allele. CKO mice were further confirmed by a 400-bp PCR product for cre-recombinase, whereas a 200-bp PCR product for WT control.

**TABLE 1:** Sequence of paired primers and the expected PCR product size.

	Name	Sequence	Expected PCR product size
Pair 1	Tcf7l2_47987_F	GGA​GAG​AGA​CGG​GGT​TTG​TG	Mutant: 139; WT: 139
	CAS_R1_Term	TCG​TGG​TAT​CGT​TAT​GCG​CC	
Pair 2	Tcf7l2_47987_F	GGA​GAG​AGA​CGG​GGT​TTG​TG	Mutant: 139, 500; WT: 139
	Tcf7l2_47987_R	CCC​ACC​TTT​GAA​TGG​GAG​AC	
Pair 3	Cre_F	TCG​ATG​CAA​CGA​GTG​ATG​AG	Mutant: 400
	Cre_R	TCC​ATG​AGT​GAA​CGA​ACC​TG	
Pair 4	Control_F	CAA​ATG​TTG​CTT​GTC​TGG​TG	WT: 200
	Control_R	GTC​AGT​CGA​GTG​CAC​AGT​TT	

### Drug Administration

Due to the high risk to develop metabolic syndrome in women, we used female animals in our experiments. Female mice (2-month-old) were used in order to exclude sex differences (Li et al., 2016; Oxenkrug and Summergrad, 2020) and were randomly divided into four groups (eight per group): 1) control mice with saline (0.9%, oral gavage) for 6 weeks; 2) control mice with olanzapine (4 mg/kg/d, oral gavage) for 6 weeks; 3) Tcf7l2 CKO mice with saline (0.9%, oral gavage) for 6 weeks; and 4) Tcf7l2 CKO mice with olanzapine (4 mg/kg/d/, oral gavage) for 6 weeks. Olanzapine (Hansoh Pharmaceutical Co. Ltd., Jiangsu, China) was dissolved in saline (0.9%) at a concentration of 0.8 mg/ml, and rigorous shaking was always performed prior to oral gavage. All drugs were administered at a fixed time (9:00 a.m.–11:00 a.m.) every day.

### Body Weight Measurement and Oral Glucose Tolerance Test

Body weight was monitored at the same time (11:00 a.m.) once each week which was used to adjust drug dosage. The rate of weight gain was calculated as follows: (body weight at week 6F02D body weight at week 0)/ body weight at week 0. For OGTT, mice were deprived of food for 16 h (at the end of week 6) and administered with 2 g/kg glucose (50%) in saline through oral gavage. Blood was sampled from the tail at 0, 30, 60, and 120 min after glucose administration. Blood glucose levels were analyzed by using a glucometer (Bayer, Germany).

### Measurement of Plasma Hormones and Lipid Levels

Blood samples from the mice that were fasted for 16 h were collected by eyeball extirpating under deep anesthesia (pentobarbital sodium, Sigma). Plasma was collected after centrifugation (3,000 rpm, 10 min, 4°C) in heparin-coated microvette tubes containing EDTA. Plasma concentrations of triglycerides, total cholesterol, HDL, and LDL were determined by using an automatic biochemical analyzer (Rayto, China). Plasma insulin, proinsulin, and glucagon levels were assessed by ELISA (Mercodia).

### Western Blot Analysis

For immunoblotting, mice were sacrificed and the pancreatic proteins were extracted and sonicated in RIPA buffer containing complete protease and phosphatase inhibitors (Roche) on ice. Then, the supernatant was collected after centrifuging at 12,000 rpm for 5 min at 4°C, and the protein concentration was determined by using the BCA protein assay kit. Equal amounts of protein were fractionated by SDS-PAGE and transferred to nitrocellulose membranes. The membranes were then blocked in 5% non-fat milk for 1.5 h, followed by incubation with primary antibodies overnight at 4°C. The following primary antibodies were used: rabbit anti-Tcf7l2 (1:2000 dilution; Novus), rabbit anti-Glp-1R (1:1000 dilution; Novus), and rabbit anti-GAPDH (1:2000 dilution; Abcam). After that, the membranes were incubated with HRP-conjugated secondary antibodies (1:2000 dilution; Sigma) and developed using an enhanced chemiluminescence kit (Millipore). The density of the bands was analyzed by ImageJ.

### HE Staining and Quantifying the Adipocyte Area

Abdominal adipose tissues were removed and were fixed in 4% paraformaldehyde solution (Solarbio), embedded in paraffin wax, and sectioned at the thickness of 5 μm with a cryostat (Leica). After deparaffinization, adipose sections were stained with hematoxylin solution for 5 min and then rinsed in distilled water. Then, the sections were stained with eosin solution for 5 min. Stained adipose sections were examined and photographed with a microscope equipped with an imaging system (Nikon Eclipse E100, Japan). The size of adipocytes was quantified by ImageJ 1.51s with the following protocol: 1) selecting a fixed area size (1,060 × 900 µm) within each adipocyte image; 2) randomly selecting 30 adipocytes in the area and measuring the absolute pixel area of each cell; 3) quantifying the amount of adipocytes in each desired adipocyte size group; and 4) calculating the percentage of adipocyte size group and then making comparison.

### Immunofluorescence Staining and Calculations of Beta Cell Mass

Isolated pancreases were fixed in 4% paraformaldehyde solution for 24 h and embedded in paraffin wax. The sections (from head to tail) were cut at 5 μm thickness and deparaffinized and rehydrated followed by rinsing with 0.01% PBS (pH 7.4). The slides were incubated overnight at 4°C with rabbit anti-Tcf7l2 (1:100 dilution; Novus) and mouse anti-insulin antibody (1:300 dilution; Servicebio), or rabbit anti-GLP-1R (1:200 dilution; Novus) and mouse anti-glucagon (1:150 dilution; R&D). After a full rinse, the slides were then incubated with Cy3-conjugated goat anti-rabbit (1:300 dilution; Wuhan Google Biotechnology Co., Ltd.) or FITC-conjugated goat anti-mouse (1:400 dilution; Wuhan Google Biotechnology Co., Ltd.) secondary antibodies, followed by nuclear counterstaining with DAPI (1:1000 dilution; Servicebio). Images were captured with an inverted fluorescence microscope (Nikon ECLIPSE TI-SR, Japan). The fluorescence intensity of each slide was quantified by ImageJ 1.51s. The whole pancreas section was scanned using Caseviewer 2.0 (3D HISTECH, Pannoramic 250/MIDI, and Hungary), and the area sizes of the pancreas and beta cells were measured for all sections. A complete pancreatic section (from head to tail) of the pancreas from each animal, including representative sections of the head, body, and tail of the pancreas, was analyzed, and all islet cells in this section per mouse were counted in each group. The beta cell mass was estimated, as previously described: pancreas mass × (insulin positive area/the size of pancreas).

### Quantitative Real-Time PCR Analysis

Total RNA was isolated from the pancreas and extracted using TRIzol (Sigma), as described previously. cDNA was synthesized from total RNA (2 mg) using the M-MLV Reverse Transcriptase Kit (TAKARA, Dalian, China). Subsequently, qRT-PCR was performed using a SYBR Premix EX Taq™ Kit (TAKARA, Dalian, China) by an ABI 7500 Real-Time PCR system (7900 HT system, ABI, United Kingdom) according to the manufacturer’s instructions. The synthesized primer sequences are listed in Table 1. The following thermocycling conditions were used for qPCR: an initial step of 93°C for 120 s, followed by 40 cycles of denaturation at 93°C for 60 s, and annealing and extension at 60°C for 60 s. Expression levels were quantified using the 2^−ΔΔCq^ method and normalized to the internal reference gene U6. RT-qPCR was performed in triplicate.

### Statistical Analysis

All statistical analyses were performed by SPSS software (version 19.0), and the values were represented as mean ± SEM. The body weight and the glucose levels of OGTT at different time points were analyzed by repeated measures ANOVA. The body weight rate, lipid and hormone levels, and adipocyte area were analyzed using two-way ANOVA. The statistical significance was set at a value of *p* < 0.05.

## Results

### Olanzapine Administration Accelerated the Speed of Weight Gain in Tcf7l2 CKO Mice

**FIGURE 2:**
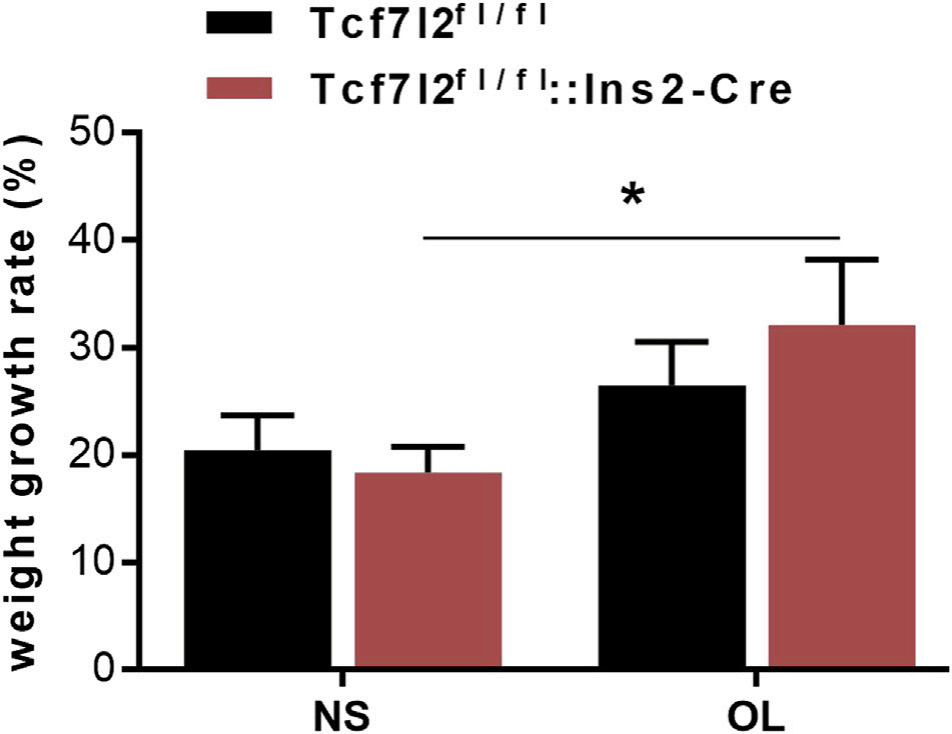
Comparison of the body weight growth rate between different treatment groups during the 6-week intragastric olanzapine administration. Tcf7l2^fl/fl^: Ins2-Cre + OL represented Tcf7l2 CKO mice challenged with olanzapine (red). Tcf7l2^fl/fl^: Ins2-Cre + NS were Tcf7l2 CKO mice administered with saline (blue). Tcf7l2^fl/fl^ + OL and Tcf7l2^fl/fl^ + NS were the control mice subjected to olanzapine (black solid line) or saline (black dotted line) treatment. All of the results were expressed as the mean ± SEM, *p < 0.05, Ol, olanzapine. NS, saline. *n* = 8 in each group.

Previous reports have demonstrated a well-established MetS-facilitating role of olanzapine in rodents (Huhn et al., 2019). To investigate the possible interplay between Tcf7l2 and olanzapine, Tcf7l2 CKO mice (Tcf7l2fl/fl:In2-cre) and littermate controls (Tcf7l2^fl/fl^) were orally gavaged with olanzapine (4 mg/kg/day) or saline for 6 weeks. The body weight was monitored every day, and the dosage of olanzapine was adjusted every week to adapt to the body weight changes as appropriate. Although the CKO mice in the olanzapine group exhibited the highest body weight gain, no difference in body weight between individual groups of mice was detected at any time during the 6 weeks. However, a robust effect of olanzapine treatment [F (1, 28) = 5.579 and *p* = 0.025] can be observed in the weight gain rate by two-way ANOVA analysis. Compared to saline treatment, Tcf7l2 CKO mice presented larger increment of weight gain and the trend of increased body weight when challenged with olanzapine [F (1, 28) = 5.409 and *p* = 0.027] (Figure 2).

### Effects of Olanzapine Treatment on Oral Glucose Tolerance Test in Tcf7l2 CKO and Control Mice

**FIGURE 3:**
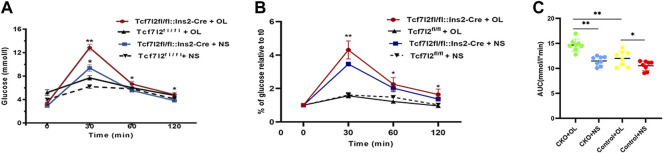
Tcf7l2 CKO mice displayed impaired oral glucose tolerance after intragastric administration of olanzapine for 6 weeks. **(A)** Oral glucose tolerance test (OGTT) on overnight fasted mice from different groups. The blood glucose level was measured at 0, 30, 60, and 120 min after glucose administration (2 g/kg body weight). Tcf7l2^fl/fl^: Ins2-Cre + OL is the Tcf7l2 CKO mouse olanzapine group (red), Tcf7l2^fl/fl^:Ins2-Cre + NS is the Tcf7l2 CKO mouse saline group (blue), Tcf7l2^fl/fl^ - + OL is control mice treated with olanzapine (black solid line), and Tcf7l2^fl/fl^ + NS is control mice treated with saline (black dotted line). *p < 0.05 and **p < 0.001. **(B)** Comparison of the percentage of blood glucose relative to fasting blood glucose at various time points among different groups. Tcf7l2^fl/fl^: Ins2-Cre + OL is the Tcf7l2 CKO mouse olanzapine group (red), Tcf7l2^fl/fl^: Ins2-Cre + NS is the Tcf7l2 CKO mouse saline group (blue), Tcf7l2^fl/fl^ - + OL is control mice treated with olanzapine (black solid line), and Tcf7l2^fl/fl^ + NS is control mice treated with saline (black dotted line). *p < 0.05 and **p < 0.001. **(C)** Area size under the blood glucose curve of four groups of mice after 6 weeks of treatment. **p < 0.001, Ol vs. NS group in Tcf7l2 KO mice, and Tcf7l2 KO mice vs. control mice in olanzapine group. *p < 0.05, Ol vs. NS group in control mice. *n* = 8 for each group. OL, olanzapine; NS, normal saline.

As Tcf7l2 is critically associated with blood glucose regulation, we then carried out the OGTT test to compare the performance of glucose tolerance between Tcf7l2 CKO and control mice after olanzapine treatment for 6 weeks. Although the time to the peak glucose all occurred at 30 min after gavage in the four groups, the CKO mice with olanzapine administration showed significantly impaired glucose tolerance as revealed by the highest peak value of plasma glucose (*p* < 0.001, red curve, Figure 3A). Compared with Tcf7l2^fl/fl^ mice, Tcf7l2 CKO mice displayed significantly higher glucose levels despite the treatment with olanzapine (red line) or saline (blue line) at 30 min (*p* = 0.034 and *p* < 0.001, respectively). In addition, the Tcf7l2 CKO mice also exhibited higher plasma glucose values at 60 and 120 min after oral olanzapine challenge (*p* < 0.05). When the blood glucose concentration was normalized against the fasting blood glucose concentration at various time points, group differences at different time points were also detected (Figure 3B). Tcf7l2 CKO mice displayed significantly higher glucose concentration than Tcf7l2^fl/fl^ mice at 30 min, regardless of olanzapine administration (all *p* < 0.001). Similarly, the Tcf7l2 CKO mice administered with olanzapine also showed higher concentration at 60 and 120 min than Tcf7l2^fl/fl^ mice (all *p* < 0.05); the concentration in the Tcf7l2 CKO mouse saline group was also higher than that of the control olanzapine group at 60 min (*p* = 0.028). For the AUC values, two-way ANOVA analysis revealed a main effect of olanzapine treatment [F (1, 28) = 31.623 and *p* < 0.001], a main effect of Tcf7l2 gene knockout [F (1, 28) = 19.043, *p* < 0.001] and an interaction effect [F (1, 28) = 4.227 and *p* = 0.049]. Tcf7l2 CKO mice in the olanzapine group exhibited higher AUC values than control mice in the olanzapine group [F (1, 28) = 20.607 and *p* < 0.001] and the Tcf7l2 KO mouse saline group [F (1, 28) = 29.487 and *p* < 0.001]. The AUC values were also significantly higher in the Tcf7l2^fl/fl^ mouse olanzapine group than the Tcf7l2^fl/fl^ mouse saline group [F (1, 28) = 6.363 and *p* = 0.0181] (Figure 3C).

### Olanzapine Treatment Altered the Expression Profile of Plasma Hormones and Lipids in Tcf7l2 CKO and Control Mice

**FIGURE 4:**
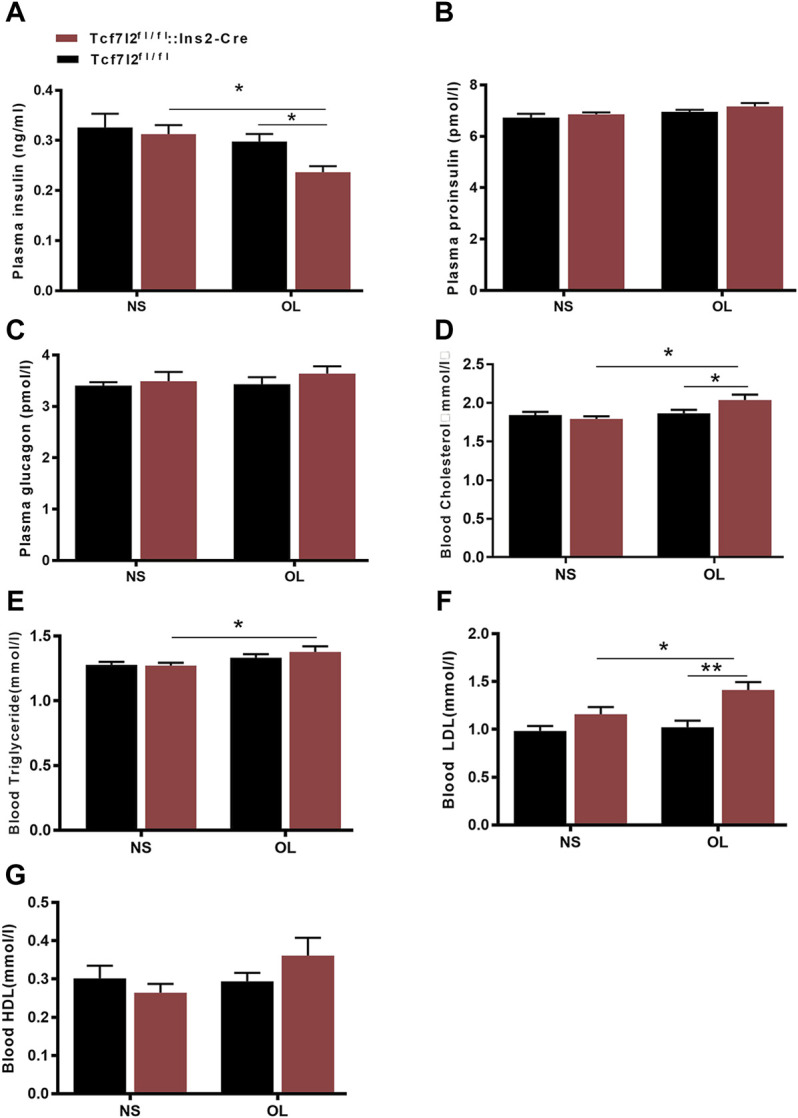
Comparison of the plasma hormones and blood lipid between different treatment groups in Tcf7l2 CKO and control mice. Tcf7l2 CKO (Tcf7l2fl/fl: Ins2-Cre, red) and control (Tcf7l2^fl/fl^, black) mice were subjected to olanzapine (4 mg/kg/day) or saline for 6 weeks. Effects of different treatment groups on insulin **(A)**, proinsulin **(B)**, glucagon **(C)**, total cholesterol **(D)**, triglyceride **(E)**, LDL-C **(F)**,, and HDL-C **(G)** at the end of 6-week treatment. All of the results are expressed as the mean ± SEM. *p < 0.05 and **p < 0.001.

As clinical administration of olanzapine has long been proposed to cause insulin resistance and dyslipidemia, we then examined the main effects of olanzapine treatment to insulin levels. As expected, the plasma insulin level was markedly lower in Tcf7l2 CKO mice with olanzapine [F (1, 20) = 7.545 and *p* = 0.012] than that in Tcf7l2 CKO mice administered with saline [F (1, 20) = 8.043 and *p* = 0.01] and Tcf7l2^fl/fl^ mice administered with olanzapine [F (1, 20) = 5.271, *p* = 0.033] (Figure 4A). However, no significant difference was observed in plasma proinsulin and glucagon between different groups (Figures 4B,C). We then measured the levels of total cholesterol (TC), high-density lipoprotein cholesterol (HDL-C), low-density lipoprotein (LDL-C), and triglyceride in serum from different groups to rule out the possibility of dyslipidemia. For the plasma TC levels, main effects of olanzapine treatment [F (1, 26) = 5.579; *p* = 0.015] and interaction effect [F (1, 26) = 4.754; *p* = 0.038] were revealed by two-way ANOVA analysis. No main effect of Tcf7l2 gene knockout [F (1, 26) = 1.507; *p* = 0.231] was confirmed. Compared to Tcf7l2 CKO mice with olanzapine, both Tcf7l2 CKO mice with saline [F (1, 26) = 11.496; *p* = 0.002] and Tcf7l2^fl/fl^ mice with olanzapine [F (1, 26) = 6.222; *p* = 0.019] showed lower TC levels (Figure 4D). Similarly, two-way ANOVA also revealed a main effect of olanzapine treatment [F (1, 26) = 6.367, *p* = 0.018] but no main effect of Tcf7l2 gene deletion [F (1, 26) = 0.359; *p* = 0.554] or interaction between Tcf7l2 deletion and olanzapine treatment in the plasma TG levels [F (1, 26) = 0.607; *p* = 0.443]. The TG levels were significantly increased in Tcf7l2 CKO mice treated with olanzapine [F (1, 26) = 5.454; *p* = 0.028] compared with Tcf7l2 CKO mice treated with saline (Figure 4E). For the plasma LDL levels, two-way ANOVA revealed a main effect of Tcf7l2 gene deletion [F (1, 26) = 15.590; *p* = 0.001] but no main effect of olanzapine treatment [F (1, 26) = 4.125, *p* = 0.053] and no interaction [F (1, 26) = 2.295, *p* = 0.142]. Moreover, the LDL levels were higher in the Tcf7l2 CKO mice with olanzapine group than the Tcf7l2fl/fl mice with olanzapine group [F (1, 26) = 15.989; *p* < 0.001) and the Tcf7l2 CKO mice with saline group [F (1, 26) = 6.286; *p* = 0.019] (Figure 4F). In line with the previous literature, no significant differences in plasma HDL levels were detected between different groups (Figure 4G).

### Effects of Olanzapine Treatment to Beta Cell Mass in Tcf7l2 CKO and Control Mice

**FIGURE 5:**
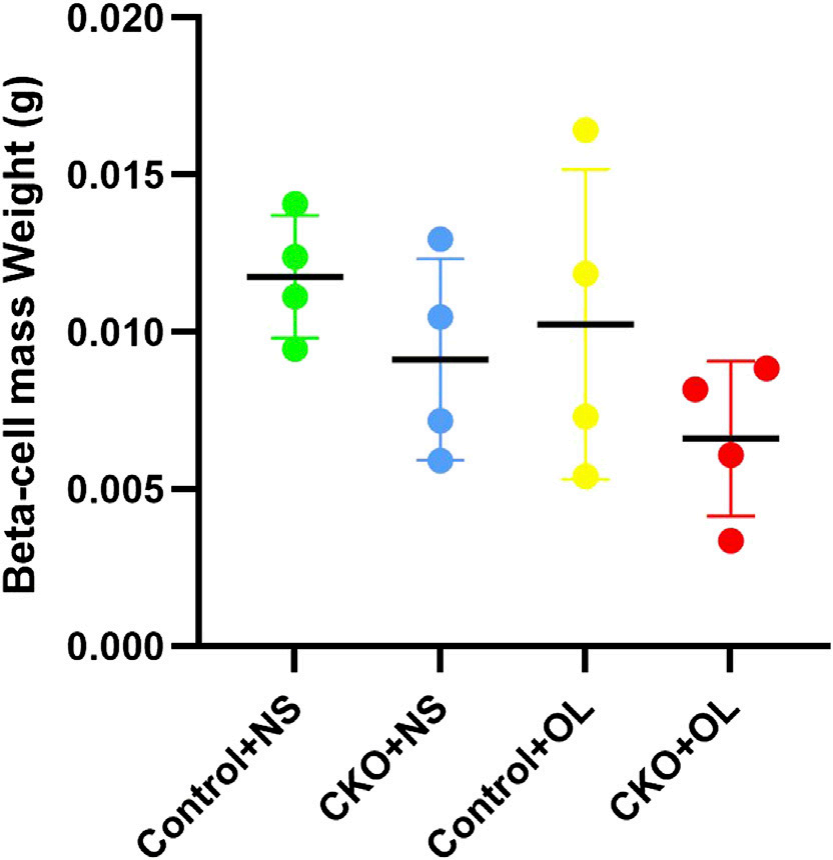
Comparison of the beta cell mass between different treatment groups in Tcf7l2 CKO and control mice. Tcf7l2 CKO (Tcf7l2fl/fl: Ins2-Cre) and control (Tcf7l2^fl/fl^) mice were subjected to olanzapine (4 mg/kg/day) or saline for 6 weeks. All of the results are expressed as the mean ± SEM. OL, olanzapine; NS, normal saline.

We further investigated whether Tcf7l2 CKO mice had altered pancreatic β-cell mass, especially after administration of olanzapine. The mass of β cells was evaluated, as previously reported (Mitchell et al., 2015), and the results are shown in (Figure 5). Although the Tcf7l2 CKO mice with olanzapine group had the least amount of pancreatic β-cell mass among four groups, no significant difference was revealed (*p* > 0.05, two-way ANOVA). This result indicates a propensity of reduced β-cell mass in Tcf7l2 CKO mice when treated with olanzapine, and this may prime the mice with the vulnerability of dysregulated β-cell function.

### Comparison of the Size and Number of Adipocytes Between Tcf7l2 CKO and Tcf7l2^fl/fl^ Mice After Olanzapine Treatment

**FIGURE 6:**
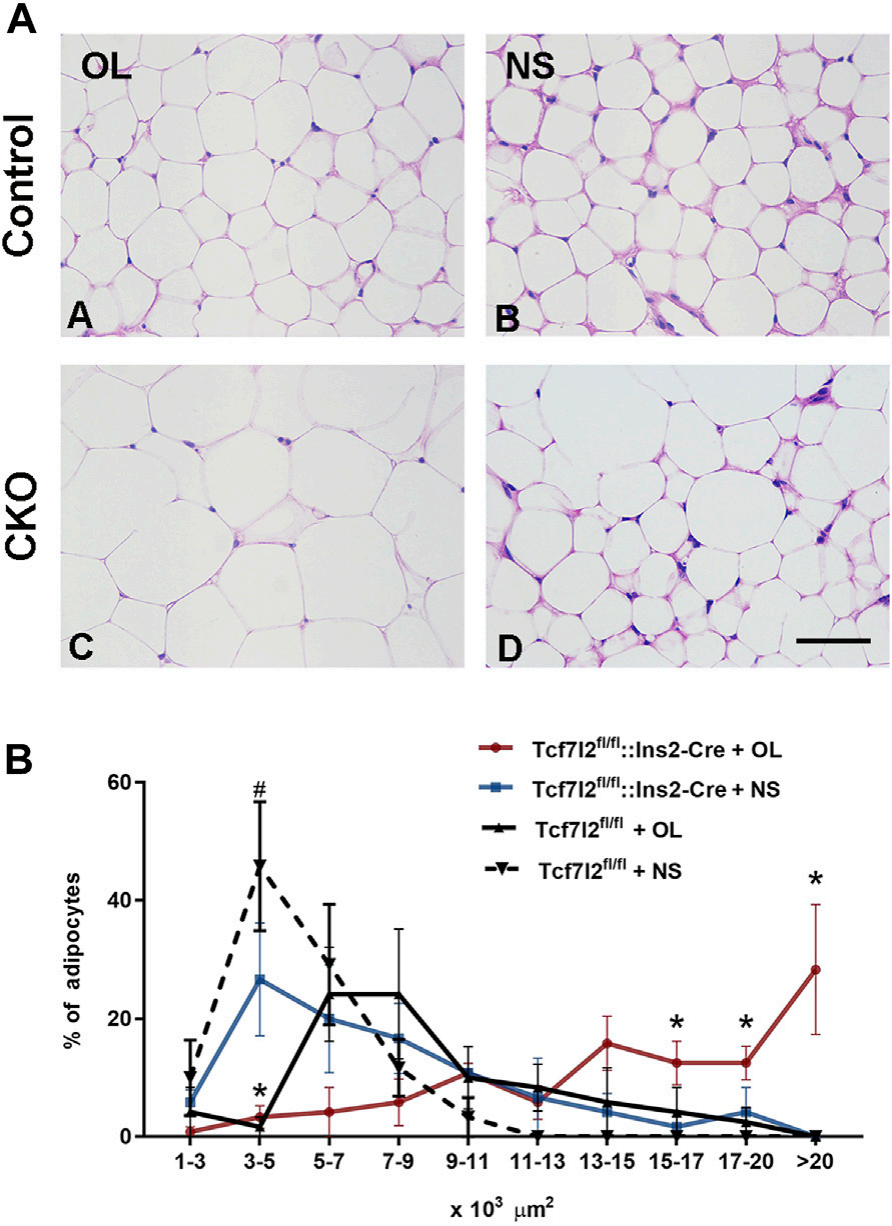
Comparison of the size and number of adipocytes between different treatment groups in Tcf7l2 CKO and control mice. **(A)** Representative images of hematoxylin and eosin (H&E)-stained abdominal adipose tissues harvested from different groups of mice after 6 weeks of treatment. A) Tcf7l2^fl/fl^ mouse olanzapine group. B) Tcf7l2^fl/fl^ mouse saline group. C) Tcf7l2 CKO mouse olanzapine group. D) Tcf7l2 CKO mouse normal saline group. **(B)** Percentage of different area sizes of adipocytes in different groups of mice after 6 weeks of treatment. *p < 0.05, Ol vs. NS group in Tcf7l2 CKO mice and Tcf7l2 CKO mice vs. Tcf7l2^fl/fl^ mice in olanzapine group. ^#^ *p* < 0.05, Ol vs. NS group in control mice. *n* = 4. Ol, olanzapine; NS, normal saline. Scale bar = 50.

Previous studies have reported that olanzapine may contribute to the hypertrophy of adipocytes in mice. In our study, we also observed higher growth rate of weight gain in Tcf7l2 CKO after olanzapine challenge, but it is unclear whether Tcf7l2 is involved in olanzapine-induced adipocyte hypertrophy. We then performed HE staining with white fat tissue and compared the size and number of adipocytes to determine fat accumulation between different groups (Figure 6A). In the saline group, the size of the adipocytes in Tcf7l2^fl/fl^ mice was roughly between 1 and 11 × 10^3^ μm^2^, and more than 40% of the cells had the size between 3 and 5 × 10^3^ μm^2^. As opposed, populations of the adipocytes in the Tcf7l2 CKO mice were larger than 11 × 10^3^ μm^2^, and less than 30% of the cells had the size between 3 and 5 × 10^3^ μm^2^ (*p* < 0.05). In the olanzapine group, most of the adipocytes of the Tcf7l2 CKO mice exhibited a cell size larger than 13 × 10^3^ μm^2^, whilst the majority of the adipocytes of Tcf7l2^fl/fl^ mice had the size between 5 and 13 × 10^3^ μm^2^. The detailed distribution pattern of the adipocyte area size is shown in (Figure 6B). For the cell size of 17–20 × 10^3^ μm^2^, variance analysis showed a main effect of Tcf7l2 gene deletion [F (1, 12) = 6.328; *p* = 0.027]. In addition, Tcf7l2 CKO mice had more adipocytes than control mice after olanzapine administration [F (1, 12) = 6.307; *p* = 0.027]. Specifically, Tcf7l2 CKO mice with olanzapine had more adipocytes with cell size over 20 × 10^3^ μm^2^ [F (1, 12) = 6.618; *p* = 0.024]. For the cells with body size between 15 and 17 × 10^3^ μm^2^, variance analysis supported a main effect of olanzapine treatment [F (1, 12) = 6.658; *p* = 0.024], and the number of adipocytes in Tcf7l2 KO mice with olanzapine is significantly larger than that of Tcf7l2 CKO mice with saline [F (1, 12) = 6.945; *p* = 0.022]. However, for the area size of 5–15 × 10^3^ μm^2^, no difference in the proportion of adipose cells among the four groups was observed (*p* > 0.05). However, for the area of 3–5×10^3^ μm^2^, variance analysis showed a main effect of olanzapine treatment [F (1, 12) = 21.051; *p* = 0.001], and both the Tcf7l2 CKO mice and control mice in the olanzapine group displayed fewer numbers of adipocytes than those treated with saline [F (1, 12) = 5.031; *p* = 0.045 and F (1, 12) = 18.026; *p* = 0.001, respectively]. However, for the area size of 1–3 × 10^3^, no difference in the proportion of adipose cells among the four groups was observed (*p* > 0.05).

### Olanzapine Treatment Decreased the Pancreatic GLP-1R Expression in Tcf7l2 KO Mice

**FIGURE 7:**
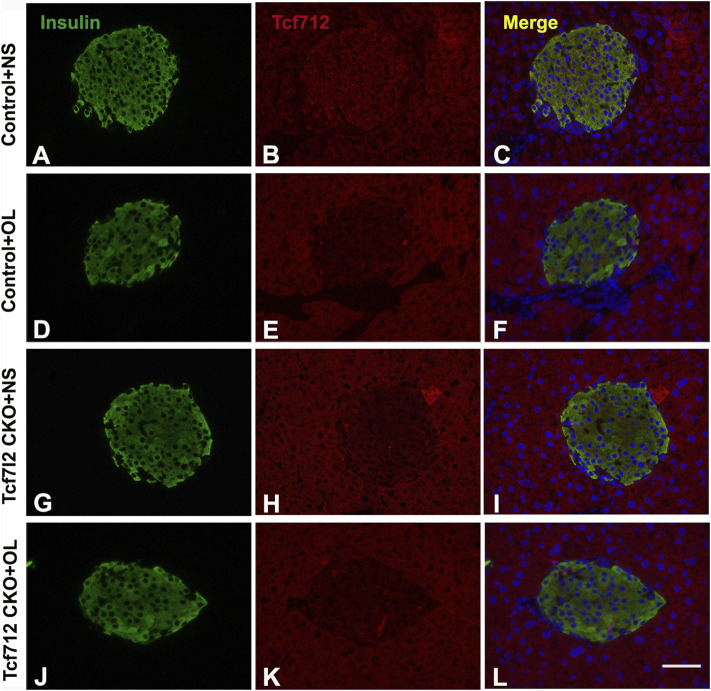
Representative immunofluorescence images of TCF7L2 in the pancreas islets of Tcf7l2 CKO and Tcf7l2^fl/fl^ mice before and after the olanzapine challenge. Immunostaining results of insulin (green) in control mice treated with saline **(A)**, control mice treated with olanzapine **(D)**, Tcf7l2 CKO mice treated with saline **(G)**, and Tcf7l2 CKO mice treated with olanzapine **(J)**. Immunostaining results of Tcf7l2 (red) in control mice treated with saline **(B)**, control mice treated with olanzapine **(E)**, Tcf7l2 CKO mice treated with saline **(H)**, and Tcf7l2 CKO mice treated with olanzapine **(K)**. Merge of insulin (green) and Tcf7l2 (red) in control mice treated with saline **(C)**, control mice treated with olanzapine **(F)**, Tcf7l2 CKO mice treated with saline **(I)**, and Tcf7l2 CKO mice treated with olanzapine **(L)**. Scale bar = 50.

**FIGURE 8:**
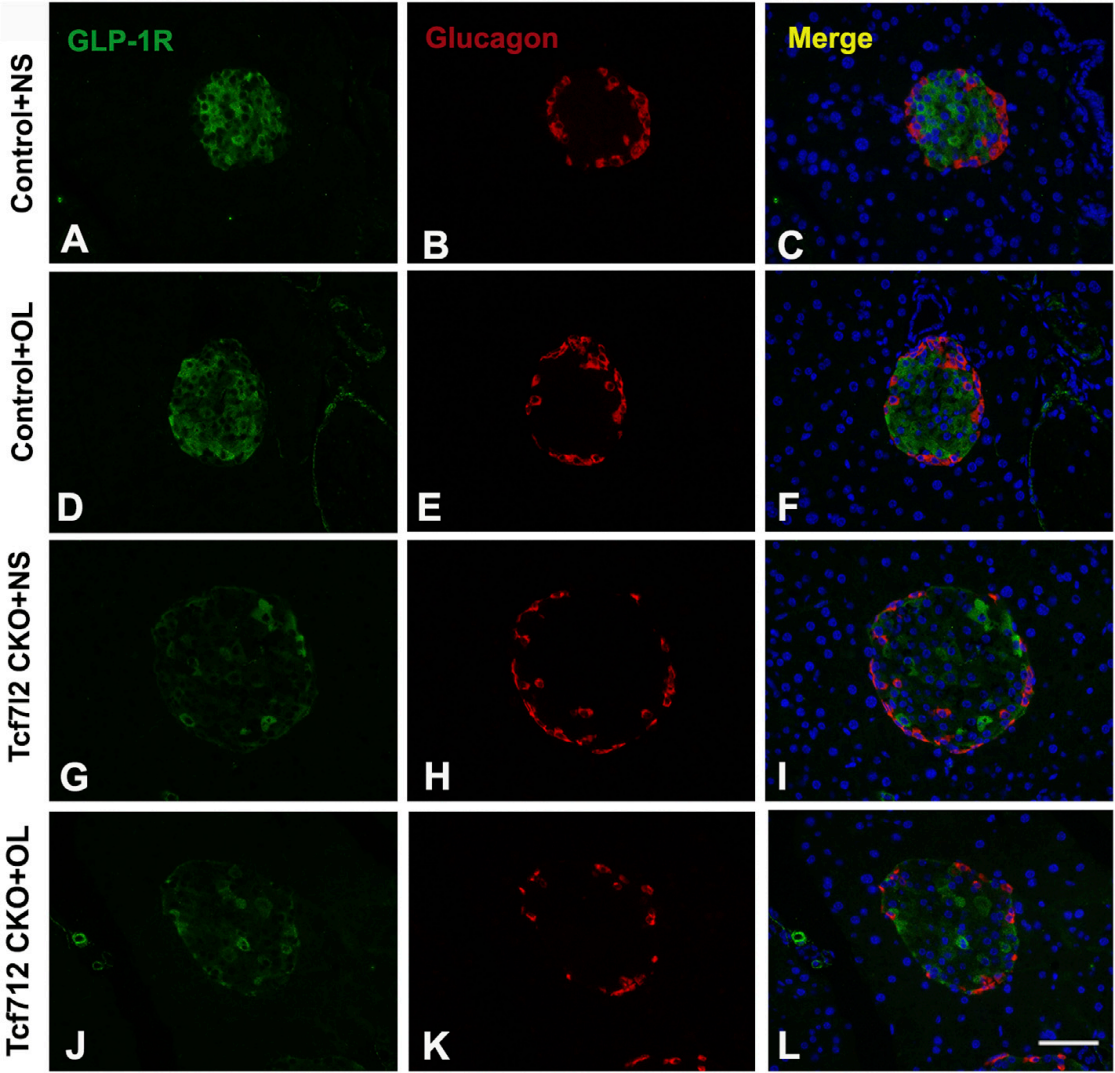
Representative immunofluorescence images of GLP-1R in pancreas islets of Tcf7l2 CKO and Tcf7l2^fl/fl^ mice before and after olanzapine challenge. Immunostaining results of GLP-1R (green) in control mice treated with saline **(A)**, control mice treated with olanzapine **(D)**, Tcf7l2 CKO mice treated with saline **(G)**, and Tcf7l2 CKO mice treated with olanzapine **(J)**. Immunostaining results of glucagon (red) in control mice treated with saline **(B)**, control mice treated with olanzapine **(E)**, Tcf7l2 CKO mice treated with saline **(H)**, and Tcf7l2 CKO mice treated with olanzapine **(K)**. Merge of GLP-1R (green) and glucagon (red) in control mice treated with saline **(C)**, control mice treated with olanzapine **(F)**, Tcf7l2 CKO mice treated with saline **(I)**, and Tcf7l2 CKO mice treated with olanzapine **(L)**. Scale bar = 50.

**FIGURE 9:**
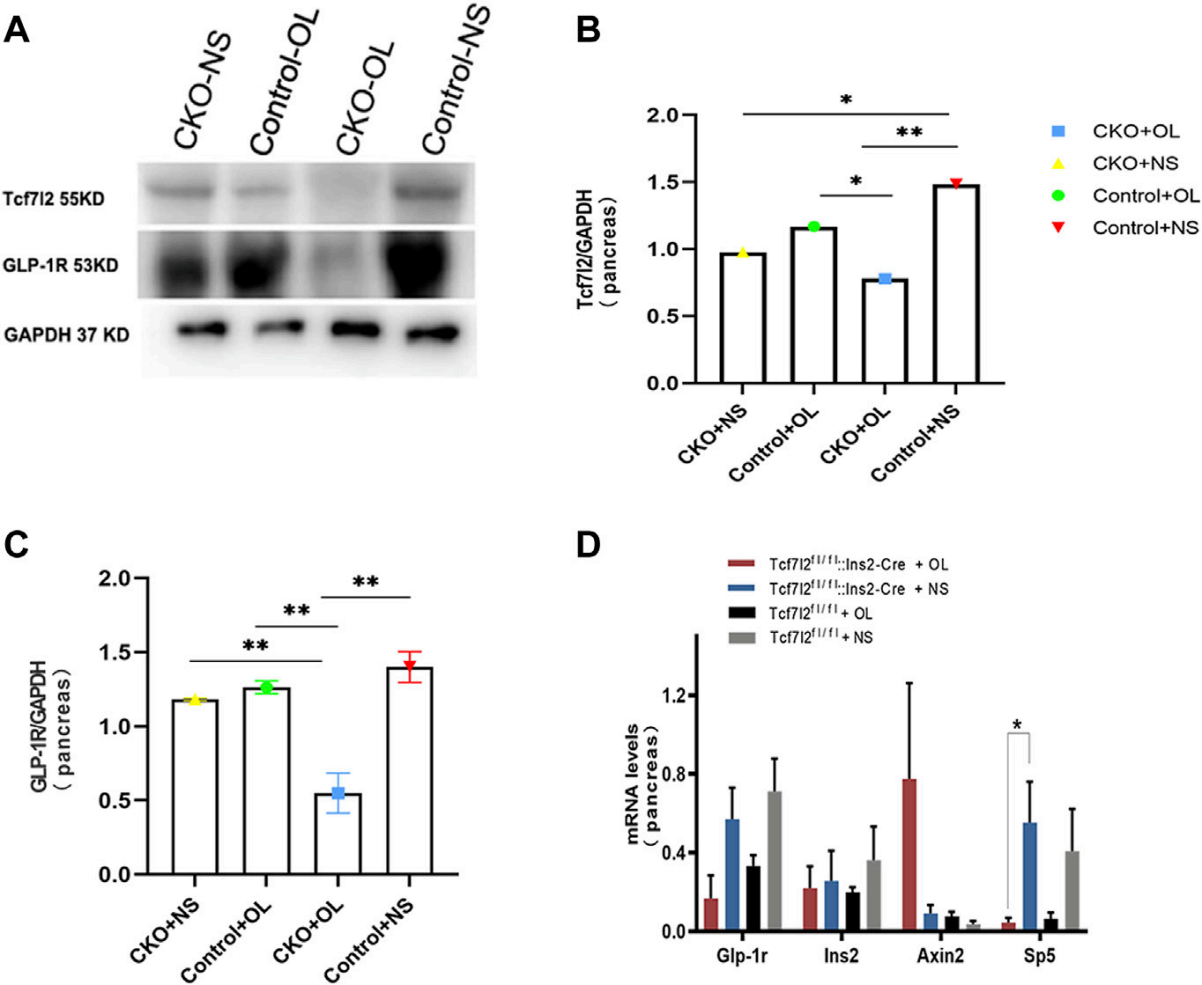
Expressions of TCF7L2 and GLP-1R in the pancreas islets of Tcf7l2 CKO and Tcf7l2^fl/fl^mice before and after the olanzapine challenge. **(A)** Protein expressions of Tcf7l2 and GLP-1R in the pancreatic tissue of Tcf7l2 CKO (Tcf7l2fl/fl: Ins2-Cre) and control (Tcf7l2fl/fl) mice. **(B)** Quantified protein expression of Tcf7l2 in the pancreatic tissue of Tcf7l2 CKO (Tcf7l2fl/fl: Ins2-Cre) and control (Tcf7l2fl/fl) mice. ^*^ *p* < 0.05 and ^**^ *p* < 0.001. OL, olanzapine; NS, normal saline. **(C)** Quantified protein expression of GLP-1R in the pancreatic tissue of Tcf7l2 CKO (Tcf7l2fl/fl: Ins2-Cre) and control (Tcf7l2fl/fl) mice. ^**^ *p* < 0.001. OL, olanzapine; NS, normal saline. **(D)** RT-qPCR analysis revealed a markedly reduction of sp5 mRNA in the pancreatic tissue of the Tcf7l2 CKO mouse olanzapine group (red) compared with the Tcf7l2 CKO mouse normal saline group (blue), *n* = 4.

Previous studies have demonstrated a strong association between impaired β-cell function and reduced Tcf7l2 and GLP-1R expression in the pancreas (Shu et al., 2009b). To test this possibility in our model, we performed immunofluorescence staining to confirm the reduced expression of Tcf7l2 and to determine the expressing profile of GLP-1R in the pancreas before and after olanzapine challenge. Compared to the control group (Figures 7A–C), reduced Tcf7l2-positive staining (Figures 7H,I,K,L) in β cells (co-stained with anti-insulin, Figures 7G,J) of Tcf7l2 CKO mouse pancreas was observed, regardless of the presence of olanzapine in the current study. Intriguingly, a reduced expression of Tcf7l2 was also observed in Tcf7l2^fl/fl^ mice subject to olanzapine treatment (Figures 7D–F), suggesting a strong inhibitory role of olanzapine to the expression of Tcf7l2 protein. Similarly, as opposed to the Tcf7l2^fl/fl^ mice with saline (Figures 8A–C) or olanzapine treatment (Figures 8D–F), an overtly decreased expression of GLP-1R was also detected in Tcf7l2 CKO mice with (Figures 8G–I) or without (Figures 8J–L) olanzapine challenge. Intriguingly, the expression of glucagon seemed unaltered (Figures 8B,E,H,K). We then run Western blotting to quantify the expression levels of Tcf7l2 and GLP-1R protein in the pancreatic tissue of the four groups (Figure 9). As expected, Tcf7l2 CKO mice displayed significantly less expression of Tcf7l2 protein which was further reduced after olanzapine treatment [F (1, 12) = 22.428; *p* < 0.001, Figure 9C]. Two-way ANOVA revealed the main effects of Tcf7l2 gene deletion [F (1, 12*)* = 19.236; *p* = 0.001] and olanzapine treatment [F (1, 12) = 28.061; *p* < 0.001], with no interaction with the expression of Tcf7l2 levels [F (1, 12) = 8.019, *p* = 0.015, Figure 9A]. Similarly, the Tcf7l2 CKO mice treated with olanzapine showed a markedly downregulated GLP-1R when compared with Tcf7l2^fl/fl^ mice treated with olanzapine [F (1, 12) = 33.041; *p* < 0.001] or Tcf7l2 CKO mice treated with saline [F (1, 12) = 26.048; *p* < 0.001] (Figure 9B). We further quantified the expressions of Sp5 and Axin2, two important components of the Wnt signaling pathway at the transcriptional level. Compared with the Tcf7l2 CKO mice with saline group, only Tcf7l2 CKO mice treated with olanzapine displayed significantly less expression of Sp5 mRNA (F = 4.845; *p* = 0.048, Figure 8D), highlighting a disturbed Wnt signaling pathway in the pancreas of the CKO mice.

## Discussion

Increasing evidence strongly suggests that a high percentage of SCZ patients display MetS and are subjected to increased risk of T2D (Postolache et al., 2019), which in turn considerably exacerbates their cognitive function. Specific psychotropic drugs like olanzapine have shown profound effects to increase MetS dysregulation, but the pleiotropy in genetic vulnerability, especially those loci leading to activated immunometabolic or endocrine pathways, also plays a pivotal role in the development of T2D and SCZ. In this study, we have determined the multifaceted roles of Tcf7l2, one of the strongest genetic determinants for T2D in humans (Hansen et al., 2011) and in the development of MetS upon olanzapine challenge in mice. We showed that mice with pancreatic β-cell–specific Tcf7l2 deletion were more vulnerable to suffer MetS after long-term administration of olanzapine. We further revealed that conditional deletion of Tcf7l2 may lead to the reduced expression of GLP-1R and a tendency of less islet β-cell mass in mouse pancreas. Our results suggest that Tcf7l2 may regulate islet cell function *via* GLP-1R in the pancreas and when defective, may enhance the deleterious effects of olanzapine and promote the occurrence of metabolic abnormalities.

How TCF7L2 orchestrates the body weight management on its own or in combination with olanzapine remains arguably inconsistent. Both Tcf7l2 dominant-negative mice and mice with pancreatic Tcl7l2 deletion displayed normal body weight (da Silva Xavier et al., 2012; Takamoto et al., 2014). Clinical research also found that TCF7L2 risk SNP allele rs7903146 is not associated with obesity, and T2D patients with TCF7L2 gene variants exhibit no heterogeneity in body weight and BMI compared to healthy controls (Ferreira et al., 2018). However, Zhang et al. (2016)have reported a close association between several TCF7L2 polymorphisms and obesity in first-episode diabetes among Chinese population. In the present study, we found that Tcf7l2 CKO mice present higher body weight growth rate when exposed to olanzapine. This is consistent with recent clinical observation that healthy Chinese people with TCF7L2 rs7093146 have larger increment of weight gain when treated with olanzapine (10 mg/d) (Li et al., 2017). These results strongly indicate that TCF7L2 is highly likely to be involved in the body weight management regulated by olanzapine.

In line with previous studies (Minet-Ringuet et al., 2007; Hou et al., 2018), our results also confirmed that chronic administration of olanzapine in mice can lead to significant adipocyte hypertrophy. This effect is even more prominent after Tcf7l2 deletion. In our case, Tcf7l2 CKO mice have more adipocytes with an area size over 17 × 10^3^ μm^2^ after continuous exposure to olanzapine for 6 weeks. Consistently, Yang et al. 2007 have shown that olanzapine induces triacylglyceride accumulation and promotes SREBP-1-related adipogenesis in differentiating 3T3-L1 preadipocytes. In addition, Minet-Ringuet et al. (2007) have revealed that adipocytes derived from olanzapine-treated rats displayed reduced lipolytic activity and increased fatty acid synthase activity, thus contributing to the excess of weight gain (Minet-Ringuet et al., 2007; Yang et al., 2007). Our study further revealed that Tcf7l2 CKO mice display significantly hypertrophic adipocytes in the presence of olanzapine, indicating a synergic effect of genetic deficit and stress. In fact, Tcf7l2 can inhibit adipogenic differentiation of 3T3-L1 and prevent adipogenesis, hence playing a role against olanzapine-induced fat deposition (Tian et al., 2017). Deletion of Tcf7l2 in mature adipocytes in mice leads to whole-body glucose intolerance and hepatic insulin resistance, which is concomitant with increased subcutaneous adipose tissue mass and adipocyte hypertrophy (Kaminska et al., 2012; Chen et al., 2018). Importantly, the TCF7L2 mRNA expression is downregulated in humans with impaired glucose tolerance and adipocyte insulin resistance. All these data strongly support that TCF7L2 may work closely with olanzapine to regulate adipocyte development and metabolism.

Consistent with previous reports (Ikegami et al., 2013; Li et al., 2018), the present study further confirmed overtly dysregulated glucose homeostasis and impaired glucose tolerance in mice challenged with olanzapine. Similarly, Tcf7l2-knockout mice also showed increased blood glucose levels and impaired glucose tolerance. In addition, mice with selective deletion of Tcf7l2 in β cells displayed impaired oral and intraperitoneal glucose tolerance at postnatal 8 and 16 weeks, respectively (Mitchell et al., 2015). In agreement with these data, we have revealed that olanzapine treatment for 6 weeks significantly exacerbates periphery glucose intolerance in Tcf7l2 CKO mice. Although a peripheral insulin resistance could not be fully ruled out, a previous study has revealed impaired glucose tolerance rather than insulin resistance when Tcf7l2 was specifically deleted in the pancreatic β cells which indicated that the impairment of glucose tolerance mainly arose from decreased secretion of insulin (Mitchell et al., 2015). These studies strongly suggest that Tcf7l2 may work synergically with olanzapine to regulate glucose homeostasis, and deletion of Tcf7l2 further promotes glucose intolerance induced by olanzapine. Our data indicate that this dysregulation may be at least partially mediated by dysfunctional GLP-1 signaling in Tcf7l2 CKO mice.

Previous reports have shown that acute administration of olanzapine has no effects on plasma insulin and glucagon levels in mice (Savoy et al., 2010; Ikegami et al., 2013). Interestingly, deletion of the Tcf7l2 gene in mouse pancreatic tissue did not disturb the fasting blood insulin, proinsulin, and glucagon concentrations (Mitchell et al., 2015). As opposed, we have demonstrated that olanzapine exposure is associated with abnormal blood insulin secretion in mice with pancreatic-specific Tcf7l2 gene deletion but not in wild-type mice. These data strongly suggest that dysfunctional Tcf7l2 may prime the mice with the vulnerability to glucose metabolism, and olanzapine may precipitate the abnormal secretion of insulin after Tcf7l2 deletion. Notably, CKO mice presented lower baseline blood glucose, indicating a disruption of insulin sensitivity may not be fully ruled out. Additionally, a previous study has also shown that islets isolated from 20-week-old Tcf7l2 gene-deficient mice displayed impaired glucose-stimulated insulin secretion (da Silva Xavier et al., 2012). Whether olanzapine administration further exacerbates insulin secretion in Tcf7l2 CKO mice requires further research in the future. Dyslipidemia is a core symptom of metabolic disorders. Notably, individuals with at-risk alleles of Tcf7l2 displayed abnormal concentrations of TC, LCL, HDL, or very low-density lipoprotein (VDLL) in periphery blood (Gallardo-Blanco et al., 2017), indicating a strong association of Tcf7l2 and dyslipidemia. In line with these findings, we have also uncovered that deletion of the Tcf7l2 gene could aggravate olanzapine-induced dyslipidemia in mice. These results support an important involvement of Tcf7l2 in the lipid metabolism.

It has been proposed that Tcf7l2 may modulate the pancreatic secretion *via* regulating the development of β-cells. Takamoto et al. (2014) have created several lines of Tcf7l2 dominant-negative transgenic mice and revealed significant reduced beta cell area and whole-pancreas insulin content in both the adult and newborn mice. On the contrary, utilizing tamoxifen-inducible pancreatic β-cell–specific Cre-recombinase mouse strain, Boj et al. (2012) found no differences in pancreatic islets and beta cell mass when tamoxifen was given after weaning. Interestingly, da Silva Xavier et al. (2012) produced pancreas-specific Tcf7l2-knockout mice which only displayed decreased oral glucose tolerance from 20 weeks with no altered β-cell mass. As opposed, they have observed 30% β-cell mass reduction in mice with β-cell–specific Tcf7l2 deletion (Mitchell et al., 2015). In our study, we have revealed that the CKO mice presented lower insulin level together with a trend of reduced β-cell mass. This feature was further exacerbated after the treatment of olanzapine. We assume that Tcf7l2 may predispose β cells to the risk of olanzapine-induced MetS, and a normal expression of Tcf7l2 can alleviate the negative effects of olanzapine and help the maintenance of proper pancreatic function and glucose homeostasis. To further reveal how Tcf7l2 affects the mass of β cells at different developmental stages, a longitudinal study utilizing the tamoxifen-inducible pancreatic β-cell–specific Cre-ER mouse strain shall be warranted in the future.

The glucagon-like peptide-1 (GLP-1) is a multifaceted hormone with broad pharmacological potential. In pancreas, it can enhance β-cell proliferation, inhibit apoptosis, and promote insulin transcription and biosynthesis (Lee et al., 2018). GLP-1 receptor agonists are successfully in clinical use for the treatment of type 2 diabetes. Intriguingly, we have detected a synchronized downregulation of Tcf7l2 and GIP-R in the CKO mice after olanzapine administration, indicating a functional synchronization between Tcf7l2 and GIP-1. We also determined the expressions of Axin and Sp5, two important components of the Wnt signal pathway. Consistent with the previous report (Boj et al., 2012), we did not detect any change in the Axin expression among different groups. However, a significantly reduced mRNA expressing level was revealed in the Tcf7l2 KO mice challenged with olanzapine, highlighting a close involvement of the Wnt signaling pathway.

This study has several limitations. For example, only a single dose of olanzapine was used, and thus, dosage-dependent effects could not be fully ruled out. Meanwhile, we did not monitor the concomitant insulin concentration during the OGTT because of the limited blood withdrawn from the vein of live mice. Another issue is that *ex vivo* insulin secretion experiments were not performed. Therefore, it is difficult to fully characterize the insulin secretion in absence of Tcf7l2 and in response to olanzapine treatment. In addition, although we have detected an overt reduction of GIP-R in the pancreas of the CKO mice, a detailed cellular and molecular mechanism still needs to be established.

In summary, our study illustrates that pancreatic Tcf7l2-knockout mice were more vulnerable to suffer metabolic abnormalities after olanzapine administration. This impairment may be mediated by the reduced expression of GLP-1R. Tcf7l2 can potentially protect pancreatic β-cell, maintain glucose homeostasis, and thus alleviate MetS caused by olanzapine. Our study provides a theoretical basis for proper application of olanzapine at clinics.

## Data Availability

The original contributions presented in the study are included in the article/Supplementary Material; further inquiries can be directed to the corresponding authors.
